# Hospital Presidents and Hospital Indebtedness

**Published:** 1909-10-30

**Authors:** 


### HOSPITAL PRESIDENTS AND HOSPITAL INDEBTEDNESS.

It is usual to regard the office of President of a hospital as a nominal position which carries dignity without direct responsibility. For this post it is customary to seek a member of the Royal Family or someone of high position in the country or in the district which the particular hospital serves. The present times are, however, extraordinary, and the financial position of institutions, mainly dependent upon voluntary support, was never more anxious than at the present moment. Parliamentary action and the trend of legislation in recent years have been in directions which are calculated to destroy rather than to create sympathy between the classes. This is a matter of grave concern to all who have the best interests of their country at heart, and no one who is really a statesman can regard with satisfaction any policy but one which cements rather than disturbs good feeling and co-operation amongst all classes of people. This is one reason why the organisation of the League of Mercy, resting as it does upon the principle of personal service in the cause of the sick, is one of paramount importance to the welfare and financial strength of the great voluntary hospitals of the United Kingdom.

In a report which we published on the Chichester Infirmary recently, attention was called to the action of King Edward in pointing out to the governors of that institution, when they applied for the privilege of incorporating the word "royal" in its title, that this institution was in debt to the extent of some £2,000, and that to be "royal" an institution must be without debt. This action has attracted much notice, for it emphasises a sound principle in hospital finance. The presidents of some of the hospitals have been looking into the accounts, and they have found not only debts, but a tendency on the part of some committees to undertake light-heartedly extensions of the work and the opening of additional beds without first obtaining the revenue to defray the cost of maintenance.

A notable example, and one which we personally hope will be largely followed, has been set by Her Royal Highness the Duchess of Albany. She is President of several funds and institutions, and she has made a stand against the system of incurring liabilities without the means in hand to meet them, and has set her face like a rock against the accumulation of debt everywhere. Of course it entails much firmness and some courage on the part of a president to adopt the course which has led to such important results in the case of Her Royal Highness. To point this out is but to emphasise the value of a president who declines to be associated with any voluntary hospital unless adequate financial care is exhibited in the policy of its managers.

We believe that this notable example will be imitated by other presidents who are worthy to hold the presidency of a great hospital. Should this prove to be the case it must result in preventing in future the issue of appeals for money not based upon audited figures and facts which are incontrovertible. In any case, every hospital committee may properly consider the bearings of the action of the Duchess of Albany, and so do their part to enforce business principles by placing the financial affairs of their institution upon a sound business basis. We heartily congratulate Her Royal Highness upon the adoption of this sound policy, and are confident that the remarkable success which has attended her courageous action will produce many imitators, to the great advantage of the voluntary hospitals all over the country.

